# Interstitial Cells of Cajal and P_2_X_3_ Receptors at Ureteropelvic Junction Obstruction and Their Relationship with Pain Response

**DOI:** 10.3390/jcm13072109

**Published:** 2024-04-04

**Authors:** Dominika Borselle, Maciej Kaczorowski, Bartosz Gogolok, Dariusz Patkowski, Marcin Polok, Agnieszka Hałoń, Wojciech Apoznański

**Affiliations:** 1 Department of Pediatric Surgery and Urology, Wroclaw Medical University and Hospital, Borowska 213, 50-556 Wroclaw, Poland; bartosz.gogolok@umw.edu.pl (B.G.); dariusz.patkowski@umw.edu.pl (D.P.); wojciech.apoznanski@umw.edu.pl (W.A.); 2 Department of Clinical and Experimental Pathology, Wroclaw Medical University and Hospital, Borowska 213, 50-556 Wroclaw, Poland; maciej.kaczorowski@umw.edu.pl (M.K.); agnieszka.halon@umw.edu.pl (A.H.); 3 Department of Pediatric Surgery and Urology, Collegium Medicum—University of Zielona Gora, 65-417 Zielona Gora, Poland; m.polok@inm.uz.zgora.pl

**Keywords:** hydronephrosis, purinergic signaling, interstitial cells of Cajal, pyeloplasty, ureteropelvic junction obstruction, congenital hydronephrosis

## Abstract

**Introduction**: Etiopathogenesis and the symptomatology of ureteropelvic junction obstruction (UPJO) in the pediatric population has not yet been definitely clarified, suggesting a multifactorial nature of the condition. The aim was to analyze the association between the number of Interstitial Cells of Cajal (ICCs), as well as P_2_X_3_ receptors in ureteropelvic junction (UPJ) and the pain response in pediatric patients with hydronephrosis. **Methods:** 50 patients with congenital hydronephrosis underwent open or laparoscopic pyeloplasty at one of two departments of pediatric surgery and urology in Poland. Patients were divided into two groups according to the pain symptoms before surgery. A total of 50 samples of UPJ were obtained intraoperatively and underwent histopathological and immunohistochemical (IHC) analysis. Quantitative assessment of ICCs was based on the number of CD117(+) cells of adequate morphology in the subepithelial layer and the muscularis propria. Expression of P_2_X_3_ receptors was evaluated as the intensity of IHC staining. **Results:** Patients with hydronephrosis and accompanying pain were on average 60 months older (77 vs. 17 months) than children with asymptomatic hydronephrosis (*p* = 0.017). Symptomatic children revealed higher numbers of ICCs in both the subepithelial layer and in the lamina muscularis propria. In particular, symptomatic patients aged 2 years or more exhibited significantly higher numbers of ICCs in the subepithelial layer. Significant differences in the distribution of ICCs between the subepithelial layer and the lamina muscularis propria were observed in both groups. Expression of P_2_X_3_ receptors was limited to the urothelium and the muscle layer and correlated between these structures. There was no relationship between pain response and the expression of P_2_X_3_ receptors. **Conclusions:** ICCs and P_2_X_3_ receptors may participate in the pathogenesis of UPJO and in the modulation of pain response to a dilatation of the pyelocaliceal system. Explanation of the role of ICCs and P_2_X_3_ receptors in propagation of ureteral peristaltic wave and the modulation of pain stimuli requires further studies.

## 1. Introduction

Congenital anomalies of the kidney and urinary tract are a spectrum of defects that may be a considerable cause of chronic kidney disease in childhood and adulthood. One of the most common forms of this spectrum is congenital hydronephrosis, caused by ureteropelvic junction obstruction (UPJO) [1,2]. Etiopathogenesis of UPJO has not yet been definitely clarified, and seems to be multifactorial. Current views on the mechanisms responsible point to the role of improper propagation of peristalsis through the ureteropelvic junction (UPJ) leading to its insufficiency and pyelocaliceal system dilatation [2,3,4]. A coordination of peristalsis in UPJ is widely attributed to specific pacemakers defined as Interstitial Cells of Cajal (ICCs), which have also been encountered in other organs and systems [2,3,4,5]. The prevailing theory regarding the pathogenesis of UPJO implicates the disruption of coordinated unidirectional smooth-muscle contractions, resulting in the attenuation of peristaltic waves responsible for facilitating the downward propulsion of urine from the renal pelvis to the ureter [6].

Distribution and density of ICCs may differ according to the patient’s age and the extent of pyelocaliceal system dilatation [7].

In the context of symptomatology of UPJO, pain response, correlated with the extent of pyelocaliceal system dilatation, is relatively rare, particularly among younger children [8]. On the contrary, in older children with hydronephrosis the pain in the lumbar or mid-abdominal region occurs more frequently. The potential role of purinergic transmitters and P_2_X_3_ receptors in the initiation and modulation of response to pain stimuli in the urinary system has been accentuated and widely discussed previously [9,10,11,12,13]. 

The purpose of this study was to analyze the association between the number of ICCs, as well as the expression of P_2_X_3_ receptors in UPJ and the pain response in the pediatric population with UPJO. Our investigation provides insight into potential mechanisms contributing to the propagation of a peristaltic wave and the modulation of pain in UPJ. 

## 2. Materials and Methods

A population of pediatric patients with congenital hydronephrosis underwent open or laparoscopic pyeloplasty at one of two departments in Poland: the Department of Pediatric Surgery and Urology of Wroclaw Medical University and Hospital or the Department of Pediatric Surgery and Urology of Collegium Medicum—University of Zielona Gora, between December 2019 and July 2022.

The patients’ data were retrospectively reviewed from medical documentation. The patients’ characteristics were collected and included the following: status at admission to the department, the patient’s history with symptom occurrence and prenatal information if available, surgical treatment details selected from the operative protocols, imaging examination assessment, post-operative outcomes, and long-term follow-up. 

Hydronephrosis was diagnosed during the routine ultrasonography, incidentally in the imaging examination performed for other reasons, or due to the symptomatology. The symptomatology included pain in older children or its behavioral equivalents in the younger children evaluated by the caregivers—crying, breathing patterns, irritability, sleeplessness, facial expression, limb movements and muscle tone [14,15,16]. The patients were then divided into two groups: symptomatic and asymptomatic. We also differentiated two subgroups according to age: below 2 years and equal to and above 2 years. The data included information on prenatal pyelocaliceal-system dilatation information from routine ultrasonography.

The indications for surgery of UPJO included the following: split renal function (SRF) < 40% in dynamic renal scintigraphy, deteriorating SRF (decrease of 5–10% in subsequent examinations), deteriorating hydronephrosis, and onset of symptoms [17,18]. The surgical treatment was laparoscopic or open pyeloplasty using the Hynes–Anderson technique in all cases with or without double-J catheter stenting. 

### 2.1. Immunohistochemistry and Evaluation of P_2_X_3_ and CD117 Expression

A total of 50 samples of UPJ were obtained intraoperatively and analyzed at the Department of Clinical and Experimental Pathology, Wroclaw Medical University, by a surgical pathologist (MK). First, the samples were fixed in formalin, and subsequently underwent automated processing and embedding in paraffin. Tissue sections were stained immunohistochemically (IHC) with antibodies to the P_2_X_3_ receptor (dilution 1:50, rabbit polyclonal, #17843-1-AP; Proteintech, Manchester, UK) and CD117 (dilution 1:100, rabbit polyclonal, #A4502; DAKO). Reactions were processed automatically with the use of PT Link pretreatment module (DAKO, Santa Clara, CA, USA) and Autostainer Link 48 (DAKO) and visualized with diaminobenzidine. FLEX Rabbit Negative Control, Ready-to-Use (Agilent DAKO, Santa Clara, CA, USA) in place of primary antibodies was used for negative controls. Moreover, additional sections were stained with hematoxylin and eosin (HE) to better visualize tissue morphology. 

In the case of P_2_X_3_, due to a consistently diffuse pattern of staining in the urothelium and smooth muscle cells (see Results), assessment of antigen expression was based on reaction intensity: 0—no expression, 1—weak expression, 2—moderate expression, and 3—strong expression.

**Figure 1:**
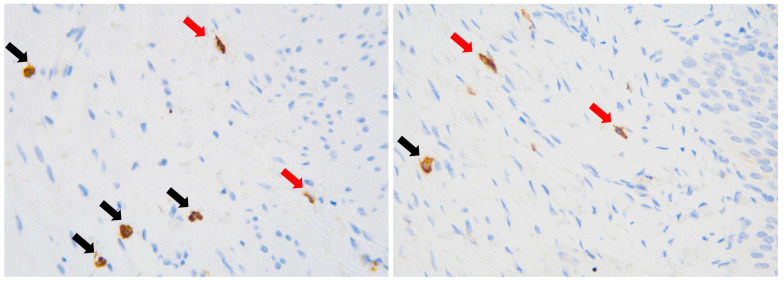
Immunohistochemical visualization of CD117(+) cells, magnification ×400. Red arrows—cells with morphology of Cajal-like cells. Black arrows—mast cells, excluded from analysis.

Quantitative assessment of ICCs was based on the number of CD117(+) cells in the submucosal layer and the muscularis propria. Ten high-power fields (HPF—field of vision with 400× magnification) were assessed for each case and each layer. Importantly, only oval and spindle cells with extended nuclei (morphology of ICCs) were counted among CD117-expressing cells, while round or oval cells with round nuclei (morphology of mast cells) were excluded (Figure 1). 

**Figure 2:**
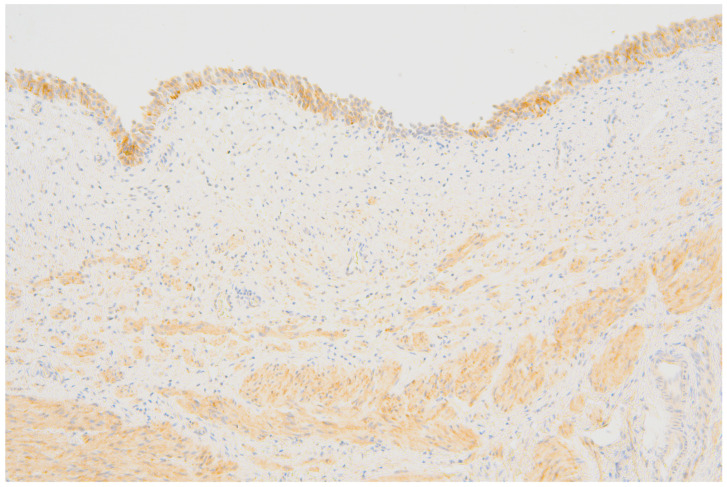
Transitional epithelium, as well as smooth muscle cells of the lamina muscularis mucosae and the muscularis propria reveal a diffuse, moderately intense expression of P_2_X_3_.

The evaluation of P_2_X_3_-receptor expression involved an intensity of IHC reaction in the 4-grade scale: 0—no expression, 1—poor expression, 2—moderate expression, and 3—intensive expression (Figure 2). 

### 2.2. Statistical Analysis 

Statistical analysis was performed in STATISTICA v. 13.3 (TIBCO Software Inc., Palo Alto, CA, USA). Verification of the normality of quantitative variables was performed using the Shapiro–Wilk test. Qualitative variables were reported as mean values ± standard deviation and as median and inter-quartile ranges, while categorical variables were reported as numbers (*n*) and percentages (%). Quantitative variables were compared using the Mann–Whitney *U* test, while categorical variables were compared using the chi-square test or Fisher’s exact test. The Wilcoxon test was used to compare two dependent groups. The Spearman’s rank correlation coefficient and the Pearson’s linear correlation coefficient were used to assess dependence strength between two features differing from normal distribution. *p*-values below 0.05 were considered statistically significant. 

This study was approved by the Ethics Committee of the Medical University in Wroclaw with the Approval Code of 779/2012, 780/2012 and 781/2012.

## 3. Results

The study population involved 50 patients: 9 (18%) females and 41 (82%) males aged between 2 months and 15.75 years. Median patient age was significantly higher in symptomatic patients (Table 1).

For further analysis, the population was stratified into two patient groups based on median age: aged under 2 years, and 2 years and above. 

### 3.1. The Number of ICCs

Symptomatic children above 2 years of age revealed significantly higher numbers of ICCs in the subepithelial layer compared to asymptomatic patients (Table 2). In the entire study cohort, symptomatic children also had elevated numbers of ICCs in the subepithelial layer compared with the asymptomatic group; nevertheless, this difference did not achieve statistical significance.

**Figure 3:**
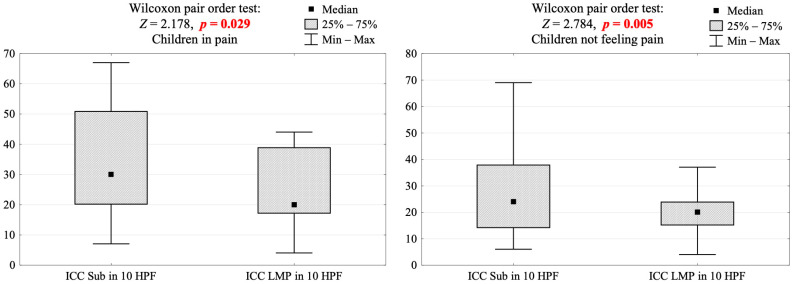
Comparison of the number of ICCs between subepithelial layer (Sub) and proper muscular layer (LMP) in 10 high-power-fields (HPF) in the population of children with or without pain response. Me [Q1; Q3]—median, lower quartile and upper quartile and the results of Wilcoxon pair-order test. Z-score test statistic value converted into *p*-value.

Significant differences in the distribution of ICCs were observed between the subepithelial layer and the lamina muscularis propria in both symptomatic and asymptomatic patients (Figure 3). Notably, these differences were accentuated in patients with pain response. It is essential to acknowledge that the cohort displaying pain response was comparatively smaller in size.

### 3.2. The Expression of P_2_X_3_ Receptors

The expression of P_2_X_3_ receptors was limited to the urothelium and smooth muscle cells of the UPJ. There were no significant differences between the expression of P_2_X_3_ receptors in these compartments (Table 3).

**Figure 4:**
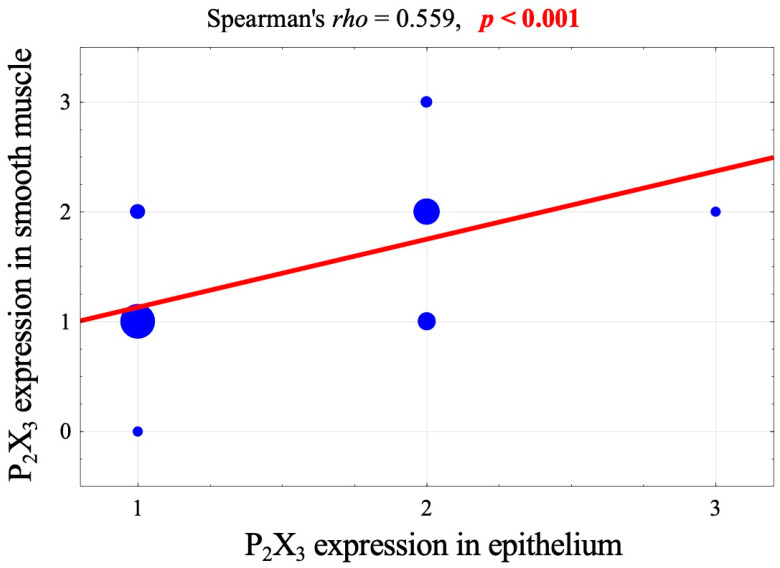
Correlation diagram of the intensity of P_2_X_3_-receptor expression in the epithelium and the muscular layer and a value of Spearman correlation coefficient and its significance.

However, a strong, significant correlation was observed between the expression of P_2_X_3_ receptors in these two layers (Figure 4).

There were no significant differences between pain response, the patient’s age, and the intensity of P_2_X_3_ receptors’ expression in the epithelium or the muscular compartment (Table 4).

## 4. Discussion

The aim of this study was to analyze the association between the number of ICCs and the expression of P_2_X_3_ receptors in UPJ and the pain response or its equivalents in pediatric patients with hydronephrosis.

The etiology and mechanisms leading to congenital hydronephrosis seem to be multifactorial, likely functional, and associated with pathophysiological pathways. Moreover, a diversity in symptomatology may be related to particular modulators participating in the response to pain stimulation and developing with age [9]. It is not yet clearly explained whether symptomatology and clinical presentation of hydronephrosis is age-dependent due to immaturity of specific sensory pathways, or whether the pathophysiology varies in different age groups. Surgical treatment of hydronephrosis involves different types of procedures with or without obstructed UPJ removal—Foley Y-V plasty [19], Fenger plasty [20], and Culp DeWeerd spiral flap [21], as well as the most popular Hynes–Anderson pyeloplasty [22]— and all of them result in an improvement in urine outflow. It may indicate a functional rather than an anatomical origin of congenital hydronephrosis. It should be noted that the term hydronephrosis is used as the meaning of the symptom, as well as to define a condition of improper urine outflow resulting in dilatation of the pyelocaliceal system.

Cajal-like cells are widely distributed in different anatomical structures, including the gastrointestinal tract and the urinary tract. ICCs are referred to as ‘pacemaker cells’, distributed in the smooth muscle layer; the lamina propria, as well as the serosa [3,4,23,24,25]. They are thought to be responsible for the initiation, propagation and coordination of peristalsis along the pyelocaliceal system and the ureter, and therefore a reduction in their density may result in changes in the ureteral motility, leading to disturbances in the passage of urine and eventually to hydronephrosis [3]. Some studies reported decreased density of ICCs in samples of UPJ in children with congenital hydronephrosis [3,4,23,26,27,28]. On the other hand, another study reported a higher density of ICCs in UPJO cases [24]. This finding could potentially be attributed to the compensatory mechanism responding to the altered peristalsis observed in UPJO [24]. However, it is important to consider that such compensatory mechanisms might diminish over time. Babu R et al. observed that changes in UPJO resembled the fetal ureter morphology with decreased ICC distribution and increased collagen-to-muscle ratio [29]. The maturation process may start at the mid ureter and its failure may lead to UPJO [29]. A meta-analysis revealed significantly lower density of ICCs in patients with UPJO compared to the healthy controls, as well as a gradual increase in ICC density with aging, in both groups [30]. Therefore, it is important to take age into consideration when comparing ICC density and distribution [30].

The distribution of ICCs may also differ just between segments of the urinary tract or even parts of the renal pelvis or the ureter wall that might be associated with the symptoms. In our study, we compared the density of ICCs in UPJ samples in two populations of patients: with and without pain before surgical treatment. The study revealed higher numbers of ICCs in the subepithelial than in the muscular layer in the subgroups; however, the differences were more noticeable in children with pain. Symptomatic children above 2 years of age revealed a significantly higher expression of ICCs in the subepithelial layer compared with the asymptomatic group. In addition, symptomatic children were significantly older than asymptomatic patients. Utilizing the median age as a reference point, we stratified the cohort into two distinct groups: those below two years of age and those equal to or above two years of age, in alignment with the existing literature [31,32]. These results may lead to the hypothesis that the expression of ICCs may increase with patient age, as well as with maturity and the development of mechanisms that provide appropriate sensation and function of the urinary tract [30]. The maturation of pathways participating in pain response may also determine the presence of the pain sensation in older children. It is possible that ICCs might play a role in modulation of pain; however, this mechanism seems to be multifactorial and more complex.

Analogically, lower numbers of ICCs and less conspicuous differences in density of ICCs between analyzed sample layers were observed in asymptomatic patients, who were also significantly younger. This observation may lead to another hypothesis of the possible association between lower ICC density and kidney dysfunction. Future studies considering the results of pre- and postoperative imaging examinations, such as ultrasonography with anteroposterior renal pelvic diameter and also scintigraphy with the evaluation of renal impairment are required to evaluate whether histopathological markers might predict the pyeloplasty outcomes. Also, a larger population of patients is necessary to verify these observations. The findings of our study were summarized in the table (Table 5).

Some authors have suggested a role of ICCs for predicting pyeloplasty outcomes with a result of >10 ICC/HPF in UPJ as the one of predictors of success [33]. On the other hand, others revealed no evidence of this hypothesis and pointed to surgical technique as more important than the histopathology of UPJ for the successful treatment of UPJO [7,34].

Another issue to discuss is the role of P_2_X_3_ receptors in pain modulation in cases of UPJO. These receptors participate in purinergic mechanosensory transduction in tubular and sac organs [9]. P_2_X_3_ receptors were reported to localize on subepithelial sensory nerves and in the smooth muscle [11,35]. In our study, their expression was observed in the surface epithelium and muscle compartment of UPJ. The intensity of staining for P_2_X_3_ in both structures was usually similar and correlated throughout the studied specimens. This strong correlation could be attributed to some variances in individual P_2_X_3-_receptor distribution linked to certain disorders, including congenital hydronephrosis, and age, rather than solely the regulation of receptors [13]. There were no differences in the intensity of P_2_X_3_-receptor expression in the context of pain response. Burnstock postulated that nociceptive mechanosensory transduction occurs at sites where the distension signal triggers the release of ATP from the epithelial cells, and this released ATP then activates P_2_X_3_ and/or P_2_X_2/3_ receptors on subepithelial sensory nerve plexuses, facilitating the transduction of sensory or nociceptive stimuli to the central nervous system [9,11]. The receptors act as fast ligand-gated ion channels and may play a role as a relevant target for some agonists or antagonists in the modulation of pain transmission and the inflammation process [9]. Nevertheless, purinergic signaling encompasses a variety of receptors and co-transmitters, rendering this transmission process intricate, particularly within the human urinary tract.

Clinicians’ ability to evaluate pain in infants is difficult and controversial, as they are non-verbal. Though a variety of validated pain-scoring systems exist, there is no standardized approach for their use [14,15,16]. The infant pain assessment involves some observable indicators: physiological, behavioral and contextual; however, they might be influenced by factors other than pain or agitation [16]. We accepted the subjective evaluation of behavioral equivalents observed by caregivers of the younger children; nevertheless, it is a limitation of the study. Another limitation is the lack of a healthy control group. Given that the presence of symptoms serves as the primary criterion for surgical intervention in hydronephrosis cases, the lack of a suitable control group hindered the evaluation of age-dependency in clinical manifestations. Therefore, we can refer our results only to the previously published data. The strong side of this study is quite a large study population treated with a homogenous technique.

The origin of congenital hydronephrosis remains controversial, as does the symptomatology in different age groups of pediatric patients with UPJO and pyelocaliceal dilatation. The mechanism seems to be multifactorial and rather functional, related to improper propagation of peristaltic wave and mechanosensory transduction. This hypothesis is supported by a similar and relatively high effectiveness of many kinds of surgical UPJ repair, as well as the fact that most patients with congenital hydronephrosis and renal function of >35% did not reveal deterioration of kidney function during follow-up [36]. In the future, there may potentially be a role for pharmacological modulation of certain purinergic receptors in UPJO [13]. In vivo studies utilizing specific antagonists or agonists-induced down-regulation for P_2_X_3_ and P_2_X_2/3_ receptors have provided evidence indicating that the inhibition of these receptors results in reduced nociceptive sensitivity across a range of various nociceptive, urological, and respiratory models [13,37,38,39]. The literature indicates the recent development of numerous selective P_2_X_3_ antagonists (such as Gefapixant); however, further investigations into the underlying pathological pathways are crucial to harness their potential in the conservative management of hydronephrosis [13,37,38,39].

## 5. Conclusions

ICCs and P_2_X_3_ receptors may participate in the pathogenesis of UPJO and in the modulation of pain response with regards to a dilatation of the pyelocaliceal system. The density of ICCs and P_2_X_3_ receptors could potentially correlate with the patient’s age and the clinical manifestation of UPJO. The explanation of the role of ICCs and P_2_X_3_ receptors in the propagation of the ureteral peristaltic wave and the modulation of pain stimuli requires further studies.

## Figures and Tables

**Table 1:** Demographic and clinical characteristics of study population. M—mean, SD—standard deviation, Me [Q1; Q3]—median, lower quartile and upper quartile.

Variable	All *n* = 50 *n* (%)	Pain Response or Its Equivalents		*p*
		Yes *n* = 11 *n* (%)	No *n* = 39 *n* (%)	
Gender:				1.000
Female	9 (18.0%)	2 (18.2%)	7 (17.9%)	
Male	41 (82.0%)	9 (81.8%)	32 (82.1%)	
Age (months):				**0.017**
M ± SD	41.7 ± 50.8	84.5 ± 64.6	29.7 ± 39.3	
Me [Q1; Q3]	18 [7; 63]	77 [25; 144]	17 [6; 35]	
Min—Max	2–189	2–187	3–189	

**Table 2:** The number of ICCs in the subepithelial layer (Sub) and the proper muscular layer (LMP-lamina muscularis propria) in 10 high-power fields (HPFs) in the entire population of children with or without pain response and also in subgroups, depending on age. Me [Q1; Q3]—median, lower quartile and upper quartile.

Variable	Patient’s Age	Pain Response or Its Equivalents		*p*
		Yes *n* = 11	No *n* = 39	
ICCs Sub in 10 HPFs	Entire population			
Me [Q1; Q3]		30 [20; 51]	24 [14; 38]	0.308
Min—Max		7–67	6–69	
ICCs LMP in 10 HPFs				
Me [Q1; Q3]		20 [17; 39]	20 [15; 24]	0.251
Min—Max		4—44	4–37	
ICCs Sub in 10 HPFs	<2 years	*n* = 2	*n* = 26	**0.006**
Me [Q1; Q3]		14 [7; 20]	24.5 [14; 40]	0.181
Min—Max		7–20	6–69	
ICCs LMP in 10 HPFs				
Me [Q1; Q3]		11 [4; 18]	17.5 [14; 26]	0.305
Min—Max		4–18	4–37	
ICCs Sub in 10 HPFs	=>2 years	*n* = 9	*n* = 13	
Me [Q1; Q3]		32 [28; 51]	22 [15; 24]	**0.025**
Min—Max		8–67	6–47	
ICCs LMP in 10 HPFs				
Me [Q1; Q3]		29 [20; 39]	21 [16; 23]	0.142
Min—Max		10–44	4–26	

**Table 3:** The intensity of staining for P_2_X_3_ receptors in the urothelial epithelium and smooth muscle cells of ureteropelvic-junction specimens and the results of the independence test. Four samples excluded from analysis—urothelium entirely denuded.

P_2_X_3_ Intensity	Epithelium n of Specimens	Smooth Muscle n of Specimens	Chi-Square Test
0	0	1	χ^2^ = 5.41 *df* = 4 *p* = 0.248
1	26	28	
2	19	19	
3	1	2	

**Table 4:** Expression of P_2_X_3_ receptors (as a 4-grade scale of intensity) in the epithelium and muscular compartment of ureteropelvic junctions in the entire population of children with or without pain response and also in subgroups, according to age. Me [Q1; Q3]—median, lower quartile and upper quartile. * cases with denuded epithelium.

Variable	Patient’s Age	Pain Response or Its Equivalents		Chi-Square Test
		Yes N = 11	No N = 39	
P_2_X_3_ epithelium intensity	Entire population			χ^2^ = 1.79 *df* = 3 *p* = 0.618
0		0 (0.0%)	0 (0.0%)	
1		7 (63.6%)	19 (48.7%)	
2		4 (36.4%)	15 (38.5%)	
3		0 (0.0%)	1 (2.6%)	
N/A *		0 (0.0%)	4 (10.3%)	
P_2_X_3_ muscle intensity				χ^2^ = 1.77 *df* = 3 *p* = 0.622
0		0 (0.0%)	1 (2.6%)	
1		7 (63.6%)	21 (53.8%)	
2		3 (27.3%)	16 (41.0%)	
3		1 (9.1%)	1 (2.6%)	
P_2_X_3_ epithelium intensity	<2 years	n = 2	n = 26	χ^2^ = 0.08 *df* = 2 *p* = 0.959
1		1 (50.0%))	13 (50.0%)	
2		1 (50.0%)	12 (46.2%)	
3		0 (0.0%)	1 (3.8%))	
P_2_X_3_ muscle intensity				χ^2^ = 1.62 *df* = 2 *p* = 0.446
1		2 (100.0%)	14 (53.8%)	
2		0 (0.0%)	11 (42.3%)	
3		0 (0.0%)	1 (3.8%)	
P_2_X_3_ epithelium intensity	≥2 years	n = 9	n = 13	χ^2^ = 3.38 *df* = 2 *p* = 0.184
1		6 (66.7%)	6 (46.2%)	
2		3 (33.3%)	3 (23.1%)	
N/A *		0 (0.0%)	4 (30.8%)	
P_2_X_3_ muscle intensity				χ^2^ = 2.18 *df* = 3 *p* = 0.536
0		0 (0.0%)	1 (7.7%)	
1		5 (55.6%)	7 (53.8%)	
2		3 (33.3%)	5 (38.5%)	
3		1 (11.1%)	0 (0.0%)	

**Table 5:** Summary of the study results in terms of ICC expression, symptomatology of UPJO and indications for surgical repair.

Symptomatic Patients	Asymptomatic Patients
Significantly older	Younger
Higher overall number of ICCs	Lower overall number of ICCs
Significant and more conspicuous differences in ICC distribution in the wall	Significant, but less conspicuous, differences in ICC distribution in the wall
Indication for surgery: symptoms with or without changes in renal function	Indication for surgery: deterioration in kidney function

## Data Availability

All authors have complete access to the study data that support the publication.
